# Radiofrequency Ablation: Honoring the Pioneers of Modern Therapeutic Innovations

**DOI:** 10.7759/cureus.72831

**Published:** 2024-11-01

**Authors:** Vladislav P Zhitny, Ryan Jannoud, Jake P Young, Brett Dixon, Brittani Bungart, Leroy Phillips, Kenneth Sutin, Jeffrey Bernstein, Mohammed Issa

**Affiliations:** 1 Department of Anesthesiology, Perioperative Care, and Pain Medicine, New York University, New York City, USA; 2 Kirk Kerkorian School of Medicine, University of Nevada, Las Vegas, Las Vegas, USA; 3 Department of Biology, The University of Utah, Salt Lake City, USA; 4 Department of Anesthesia, Critical Care, and Pain Medicine, Massachusetts General Hospital, Boston, USA; 5 Department of Anaesthesiology, Harvard Medical School, Boston, USA; 6 Department of Anesthesiology, Perioperative, and Pain Medicine, Brigham and Women's Hospital, Harvard Medical School, Boston, USA

**Keywords:** chronic pain, chronic pain management, healthcare history, historical vignette, radiofrequency ablation (rfa)

## Abstract

Radiofrequency ablation (RFA) is a minimally invasive technique used to alleviate chronic pain by destroying pain-signaling nerve fibers. This procedure offers precise and focused pain relief, serving as an alternative to surgical and pharmacological treatments for chronic conditions such as arthritis, back pain, neck pain, and nerve pain. RFA has evolved over a long period, beginning with early work on electrical currents. Significant progress has been made in its application to pain management with continual technological improvements enhancing its safety and effectiveness. As technology and research continue to progress, RFA promises even greater efficacy and safety, enhancing the quality of life for patients with chronic pain. Additionally, as the understanding of pain mechanisms advances, RFA can be utilized as a procedure to treat a wider lexicon of chronic pain pathologies. This historical narrative outlines the history and clinical origins of RFA, the pioneers that pushed the development of the field, and the treatment's path to modern implications in chronic pain relief.

## Introduction and background

Radiofrequency ablation (RFA) is a minimally invasive medical procedure that utilizes radiofrequency (RF) energy to heat and destroy targeted tissues. RF energy, a type of electromagnetic energy, is composed of radio waves with frequencies ranging from 300 kHz to 1 MHz. These frequencies are non-ionizing and subsequently do not cause ionizing radiation damage [1].

Initially developed for various medical applications, RFA has significantly evolved over the years, expanding its use in fields such as oncology, cardiology, and pain management [2]. In pain management, RFA has gained significance as a technique to alleviate chronic pain by heating and destroying all cells within a specific zone around the electrode tip. RFA is commonly used to treat chronic pain conditions such as arthritis, lower back pain, and various types of nerve pain including peripheral nerve pain, trigeminal nerve pain, and lumbar spinal nerve pain. This has been shown to be an effective and safe treatment option for many patients, particularly those with lumbar facet syndrome [3].

One of the key advantages of RFA in pain management is its ability to provide targeted and precise pain relief. Unlike medications, which can have systemic side effects and may not effectively target the pain source, RFA enables physicians to precisely identify and ablate the specific nerves causing pain, such as the dorsal root ganglia for back pain, the suprascapular nerve for shoulder pain, or the splanchnic nerves for pancreatic pain [2]. This targeted approach can lead to significant pain reduction and improved quality of life for patients who have not found relief with other treatments. Additionally, RFA is a relatively low-risk procedure with minimal complications, which may include pain at the injection site, swelling, bruising, infection, and changes in sensation. Although rare, more serious complications can occur, such as neuroma formation, paralysis, allergic reactions, vascular complications, pneumothorax, adhesions, and thermal injuries, with varying levels of severity during or after the procedure [4]. The infrequency of these serious complications makes RFA a viable option for patients seeking alternative pain management methods [5].

While RFA has shown considerable promise in various applications, it is essential to compare its success to existing techniques. In the realm of pain management, RFA has often been positioned against other interventional procedures such as spinal cord stimulation (SCS), nerve blocks, and pharmacologic therapies. Studies have indicated that RFA may offer superior long-term pain relief compared to nerve blocks, which often require repeated treatments due to their temporary nature [6]. In contrast to spinal cord stimulation, which involves the implantation of a device and ongoing management, RFA is a one-time procedure that can be more cost-effective and less invasive [7]. However, it is worth noting that RFA might not be suitable for all patients, and its success rate can vary depending on the specific condition and patient population.

It is further worth noting that RFA has applications outside the realm of pain management. In oncology, RFA has emerged as a potential alternative to surgical resection for certain tumors, particularly in patients who are not candidates for surgery due to comorbidities or the location of the tumor [8]. For instance, in the treatment of hepatocellular carcinoma (HCC), RFA has demonstrated comparable efficacy to surgical resection in small, localized tumors, offering a minimally invasive option with fewer complications and shorter recovery times [9]. However, in cases of larger or more complex tumors, RFA may be less effective than surgery, and in some instances, its use has been combined with other treatments such as chemotherapy or radiation therapy to enhance outcomes [10]. Thus, while RFA has been successful in certain contexts, it has not entirely replaced other techniques but rather serves as a complementary tool.

As research continues to demonstrate the efficacy of RFA in relieving chronic pain, RFA is likely to become an increasingly popular treatment option for patients seeking long-term pain relief without the need for surgery or ongoing medication [2,11]. In this piece, we explore the long history of RFA and highlight the pioneers that advanced the treatment modality into modernity.

## Review

RFA has a rich contemporary history marked by significant advancements in technology as outlined in Table 1. However, the utilization of RFA goes back much further than many might expect. This minimally invasive procedure utilizes RF energy to agitate the surrounding tissue, generating heat and leading to coagulative necrosis of the targeted cells [11]. Through the use of various types of RFA needles such as standard, cooled-tip, cluster, expandable, and bipolar, specific areas can be further targeted. This offers an alternative to surgery for various medical conditions. The evolution of RFA can be traced through a series of key milestones.

**Table 1:** Contemporary advancements of RFA technology RFA: radiofrequency ablation

Year	Advancement	Citation
1973	The Shealy Rhizolysis kit is standardized, with spinal needles of varying gauges and electrodes for threading to the desired RFA site.	[12]
1980s	Utilization of fluoroscopy for precise placement of needles.	[13]
1998	Pulsed waveform radiofrequency energy generators are trialed leading to increased control over focal lesion temperatures.	[14]
2001	Cooled-tip RFA electrodes are widely researched and employed, improving control over lesion size and reducing procedural complications.	[15]
2005	Incorporation of microwave frequency ablation as an alternative to RFA, offering faster treatment times and larger ablation zones.	[16]
2010	Integration of RFA with advanced imaging modalities such as MRI and CT, enabling real-time monitoring and precise targeting of lesions.	[17]
2014	Emergence of robotic-assisted RFA systems paired with various imaging modalities, enhancing procedural accuracy and enabling remote operation.	[18]

1891

**Figure 1:**
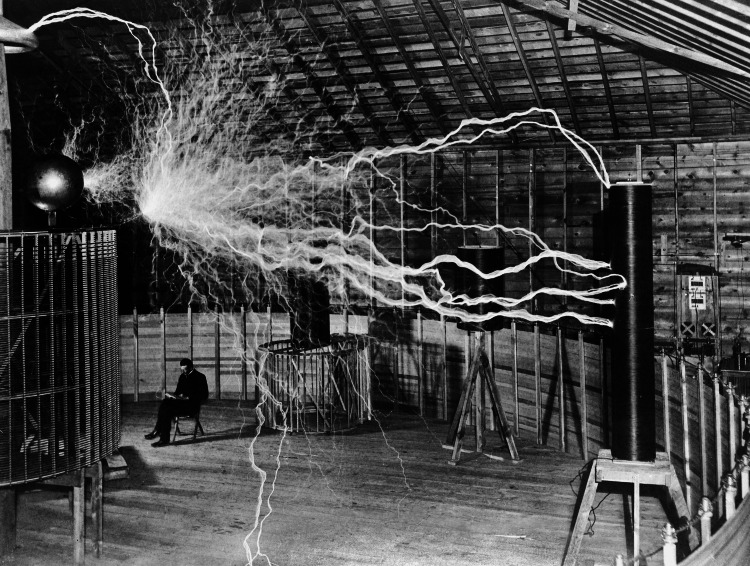
Nikola Tesla in his laboratory in Colorado Springs in December 1899 Image Source: Wellcome Collection [20]; Licence: Attribution 4.0 International (CC BY 4.0)

The transformer, which creates high frequency and high voltage currents, was invented by Nikola Tesla. Tesla, a scientist, physicist, engineer, and inventor, was the first to realize the use of electrical currents for therapeutic purposes. Tesla applied these currents, which convert electrical energy into heat, to his own body. He became the first to witness the beneficial physiological effects of this heat therapy. He patented this technology, seen in Figure 1, which would later be known as the Tesla coil. This invention laid the foundation for the use of RF energy in medical applications [19].

1931

**Figure 2:**
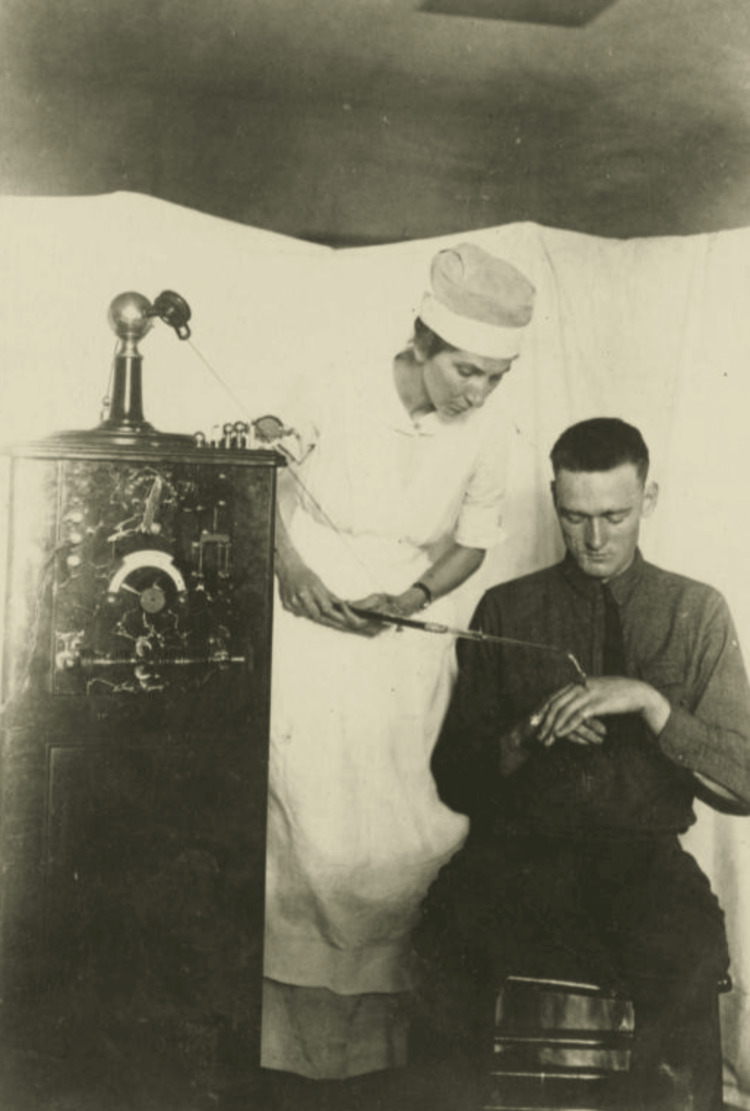
A nurse oversees the application of a fulguration device on a patient's hand, date unknown, World War I era Image Source: Wikimedia Commons [22]; licensed under the Creative Commons Attribution 2.0 Generic license

The first case of percutaneous electrode treatment for trigeminal neuralgia was successfully carried out by German surgeon, Dr. Martin Kirschner. This groundbreaking approach was built upon pain treatments used in the First World War (Figure 2) and provided a foundational method for the targeted management of nerve-related pain [21]. Kirschner's work not only advanced the understanding and capabilities of neurosurgical treatments but also left a lasting legacy in the medical field, paving the way for further innovations in RFA and other pain management technologies. His contributions remain a cornerstone in the history of medical treatments for chronic pain, reflecting a significant milestone in the evolution of modern therapeutic techniques [9].

1950s

The first radiofrequency ablation device became available on the commercial market from Dr. Sidney Aronow and Bernard Cosman. These early devices operated without water-cooling mechanisms, relying instead on standard electrodes to produce thermal lesions via controlled radiofrequency energy. The device's design aimed to achieve precise tissue ablation by generating focused heat, enabling targeted lesion formation. At this point in technological development, RFA was particularly effective for treating chronic pain and neurological conditions through disrupting pain pathways [23]. These early devices, while limited by the lack of advanced cooling mechanisms, represented a significant step forward in minimally invasive therapies, providing a novel method for creating well-defined thermal lesions.

1973

A new method for lesioning the articular nerves to the lumbar facet joints using a percutaneous radiofrequency electrode was developed by American neurosurgeon Clyde Norman Shealy. Building upon Kirscher’s work on thermal lesioning, Shealy adapted this approach for chronic low back pain. This innovation offered a new, minimally invasive treatment option for spinal pain, broadening the scope of RFA and setting the stage for further research and clinical applications in chronic pain management [12].

1990

The use of RFA for hepatocellular carcinoma was applied by Dr. Tito Livraghi, focusing on its effects on medium and large lesions in the liver. His research demonstrated RFA’s efficacy and safety as a minimally invasive treatment, offering a viable alternative for patients unsuitable for surgical resection. Livraghi’s work provided crucial evidence for the adoption of RFA in oncology, ultimately expanding its application to other tumor types and establishing RFA as a standard treatment modality in interventional oncology [24,25].

2000s

Technological advancements in RFA, such as the introduction of cooled-tip electrodes and image-guided systems, enhance the safety and efficacy of the procedure. The cooling of the electrode tip enables a more controlled and predictable heating of the tissue, reducing the likelihood of overheating and tissue damage. This additional safety measure is particularly important in tissues with high blood flow, such as the liver, or in highly selective nervous ablation procedures, like those targeting specific nerve roots or ganglia. While these measures enhance safety and efficacy, they also introduce complexities and higher costs that limit accessibility [26].

**Figure 3:**
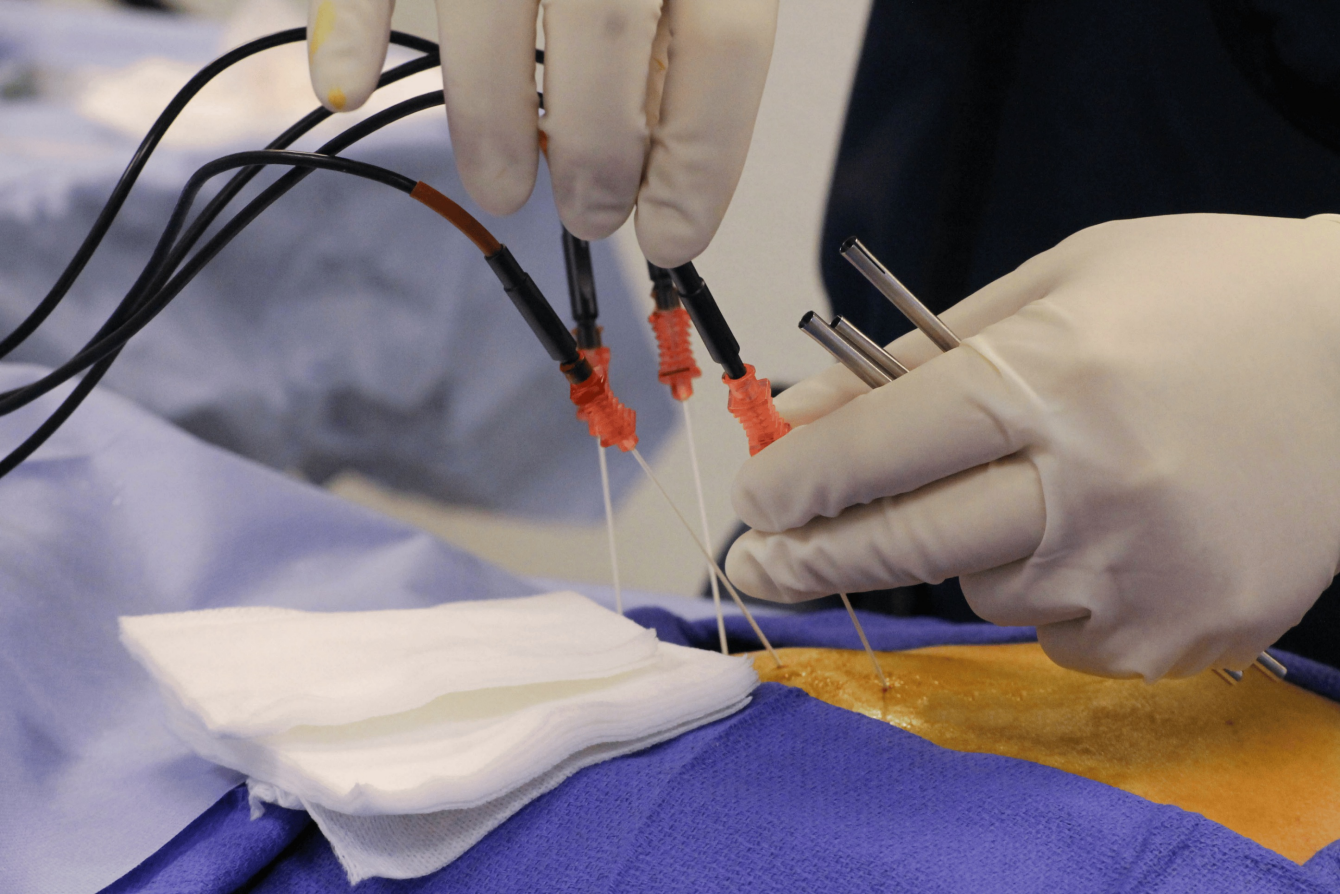
Modern radiofrequency ablation procedure for chronic pain Image Source: Wikimedia Commons [29]; licensed under the Creative Commons Attribution-Share Alike 4.0 International license

Additionally, the development of image-guided systems has enhanced the precision of ablation procedures. Through the use of ultrasound, CT, MRI, or fluoroscopy, clinicians can achieve real-time visualization of the target tissue, facilitating more accurate placement of electrodes [27]. This precision is further augmented by performing electrical stimulation testing before ablation, ensuring that the treatment zone does not impact unintended structures such as major blood vessels, nerves, or other critical anatomy. This capability allows for more precise pinpointing of the therapeutic region, which reduces the risk of complications and enhances patient safety (Figure 3). These advances have expanded the clinical applications of RFA, making it a viable option for treating various conditions, including cardiac arrhythmias, chronic pain syndromes, and a range of solid tumors, including those in the liver, lung, kidney, and bone [27,28].

In cardiac applications, for example, RFA has become a foundational treatment for arrhythmias, with the cooled-tip technology allowing for deeper and more controlled lesions, critical for targeting aberrant electrical pathways in the heart. This has led to higher success rates and a reduction in the recurrence of arrhythmias [30]. In oncology, image-guided RFA has allowed for the precise targeting of tumors, offering a minimally invasive alternative to surgery, particularly for patients who are poor surgical candidates. For example, in gastroenterology, endoscopic ultrasound-guided RFA (EUS-RFA) has emerged as a promising tool for treating pancreatic pathologies, including pancreatic neuroendocrine tumors and cystic lesions [31]. EUS-RFA offers precise ablation with minimal invasiveness, potentially providing a safe alternative for patients ineligible for major pancreatic surgeries. Early studies show promising efficacy with high response rates and low complication rates. Furthermore, RFA’s ability to target small tumors or metastatic lesions with precision has broadened its role in palliative care, providing symptomatic relief and improving quality of life [9].

The future of RFA and challenges

While RFA has become a mainstay in several medical specialties, its potential applications in other fields remain an active area of research. Recently, the use of RFA for chronic obstructive pulmonary disease (COPD) has gained attention, with preliminary animal studies suggesting that external radiofrequency application may enhance lung compliance and reduce COPD symptom severity [32]. This promising research hints at new therapeutic possibilities for COPD management, potentially expanding the range of pulmonary RFA procedures alongside existing techniques like bronchial thermoplasty [33].

Despite these advancements, challenges remain, including the steep learning curve for practitioners and the need for further refinement in equipment to minimize procedural complexities. As the scope of RFA continues to expand, ongoing research is focused on optimizing electrode designs and developing hybrid techniques with other therapies, such as immunotherapy or chemotherapy, to further improve outcomes in cancer treatment. These innovations not only have the potential to make RFA safer and more effective but also to broaden its accessibility and affordability across different healthcare settings [34].

## Conclusions

The history of RFA is a testament to the ingenuity and dedication of researchers, physicians, and engineers who have continually pushed the boundaries of medical technology. From its humble beginnings in the early 20th century to its current status as a versatile and effective treatment modality, the development of RFA reflects the relentless pursuit of innovation and excellence in healthcare. As technology continues to advance, there are promising prospects for further enhancing the efficacy and safety of RFA. Additionally, as new targets are identified, the applications of RFA are likely to expand, offering greater benefits in the treatment of chronic pain and further advancing the field of pain management.
